# Editorial: Nutrition Management for Chronic Kidney Disease

**DOI:** 10.3390/nu12123852

**Published:** 2020-12-17

**Authors:** Vassilios Liakopoulos, Evangelia Dounousi

**Affiliations:** 1 Division of Nephrology and Hypertension, 1st Department of Internal Medicine, AHEPA Hospital, Medical School, Aristotle University of Thessaloniki, 54636 Thessaloniki, Greece; 2 Department of Nephrology, Faculty of Medicine, School of Health Sciences, University of Ioannina, 45110 Ioannina, Greece; evangeldou@gmail.com

Chronic kidney disease (CKD) constitutes a major health problem worldwide. Patients with severe CKD and dialysis patients exhibit an incredibly high risk of death, mainly due to cardiovascular disease, which is not sufficiently explained by traditional or non-traditional, uremia-related risk factors. Nutritional disorders, with more frequent undernutrition, have been associated with poor quality of life and reduced patient survival. Optimal nutritional status remains a poorly established issue, while the nutritional management of non-dialysis, dialysis and transplanted patients is a tremendously challenging area of everyday clinical practice. The existence of other comorbidities, such as diabetes and hypertension along with CKD complications such as mineral bone disease, electrolyte abnormalities, fluid balance disorders, protein-energy wasting, and inflammatory status further complicates the management of nutrition in this heterogeneous patient population. The Kidney Disease Outcomes Quality Initiative (KDOQI) Clinical Practice Guidelines for Nutrition in CKD aim to frame nutritional assessment, and provide recommendations based on current evidence for the best practice in CKD [1]. Although there has been progress in nutritional targets in CKD, the quality of existing evidence is rather low, and important clinical topics remain unanswered. This special issue is an attempt to a holistic approach of the nutritional management of CKD along the different stages from non-dialysis CKD 1–5 patients, to patients undergoing various dialysis modalities and renal transplant recipients. The published papers deal with many interesting aspects of nutrition in both pre-dialysis and dialysis patients.

Nutritional support, nutritional habits and interventions, micronutrients, phosphate control, and avoidance of malnutrition and protein-energy wasting are of great importance for reaching optimal nutritional status in dialysis patients. Eating during a dialysis session is an issue that attracted particular attention in this special issue. Hemodialysis (HD) sessions represent a valuable opportunity to monitor nutritional status and prescribe nutritional interventions. Piccoli et al. reviewed the current evidence on intradialytic nutrition and proposed an algorithm for adapting nutritional interventions to individual patients [2]. Fotiadou and coworkers, on the other hand, suggested that anticipated nutritional benefits should always be balanced against the increased incidence of intradialytic hemodynamic instability [3].

Kiebalo et al. assessed malnutrition in peritoneal dialysis (PD) patients, reviewed the current nutritional recommendations of various societies, and provided recommendations for patient screening, assessment of malnutrition and protein-energy wasting, as well as nutritional intake and support of patients on chronic PD [4]. Kuwasawa-Iwasaki and coworkers showed that L-carnitine supplementation reduced the incidence of muscle cramps and improved hemoglobin levels, especially in long-term peritoneal dialysis and hemodialysis patients [5]. Bakaloudi et al. performed a systematic review on the effect of exercise on nutritional status of HD patients, suggesting that exercise could be beneficial for body composition and nutritional status [6]. Phosphate control is a difficult task in CKD, and many parameters should be taken into consideration including ethnic and regional eating habits. In this issue, Palafox-Serdan et al. assessed the phosphate intake of CKD patients in Mexico and provided a detailed chart about the phosphate content of different foods, most of which were traditionally Mexican. They also included charts of the phosphate content of various additives and commonly prescribed drugs to assess sources of inorganic phosphate intake [7].

Estimating nutritional intake and dietary monitoring is of utmost importance for providing proper nutritional counselling. Abdel-Nabey et al., on behalf of the French NephroTest Study Group, studied over 400 CKD patients and concluded that twenty-four-hour urine collections are feasible and represent a reliable tool for dietary monitoring and could be used in making individual recommendations for salt and protein intake [8]. Expanding on the topic of assessing nutritional parameters, Ginos and Olde-Engberink reviewed evidence on sodium and potassium intake methods based on twenty-four-hour urine collections or spot urine estimations (including spot Na/K ratio) in the general population and CKD patients, exploring suggestions that may improve test accuracy without increasing the associated burden for the patients [9].

The effectiveness of traditional medicines is always a hot topic, and Hong et al. performed a study in rats with renovascular hypertension and showed an antihypertensive effect of Gynura Divaricata (a Chinese traditional medicine) probably by modulation of the Renin Angiotensin Aldosterone System (RAAS) [10].

Uric acid was also a major focus in this issue. Floriano and coworkers used bioelectrical impedance analysis, hand grip strength tests and five repetitions of sit-to-stand tests in a cohort of 113 kidney transplant recipients and showed that uric acid (UA) levels were positively associated with muscle mass and strength, but not with functional capacity [11], while Dominguez-Zabrano et al. underlined the antioxidant properties of uric acid in HD patients [12]. In another interesting study involving living kidney donors, Oba and coworkers showed that single nephron Glomerular Filtration Rate (GFR) was directly associated with protein intake but not with sodium intake, BMI or arterial pressure [13].

The studies published under the special issue “Nutritional management for CKD” highlight the importance of nutritional interventions in all aspects of CKD and underline the need for the nutritional counselling of patients across all stages. In the era of individualized medicine, more research in the field will certainly shed light in many controversial issues and prove beneficial in the holistic approach and management of this special patient population.
